# Supplementary material for: Cre Recombinase Mediates the Removal of Bacterial Backbone to Efficiently Generate rSV40
Source: Mol Ther Methods Clin Dev. 2018 Feb 27;9:225–33. doi: 10.1016/j.omtm.2018.02.010 (PMC5948228; doi:10.1016/j.omtm.2018.02.010)
Supplement: Document S1. Figures S1–S3 and Table S1 [file mmc1.pdf]

OMTM, Volume *9*

Supplemental Information

Cre Recombinase Mediates the Removal of Bacterial Backbone to Efficiently Generate rSV40

Xiaoxia Shi, Matthew Ryan Ykema, Jaco Hazenoot, Lysbeth ten Bloemendaal, Irene Mancini, Machteld Odijk, Peter de Haan, and Piter J. Bosma

Figure S1

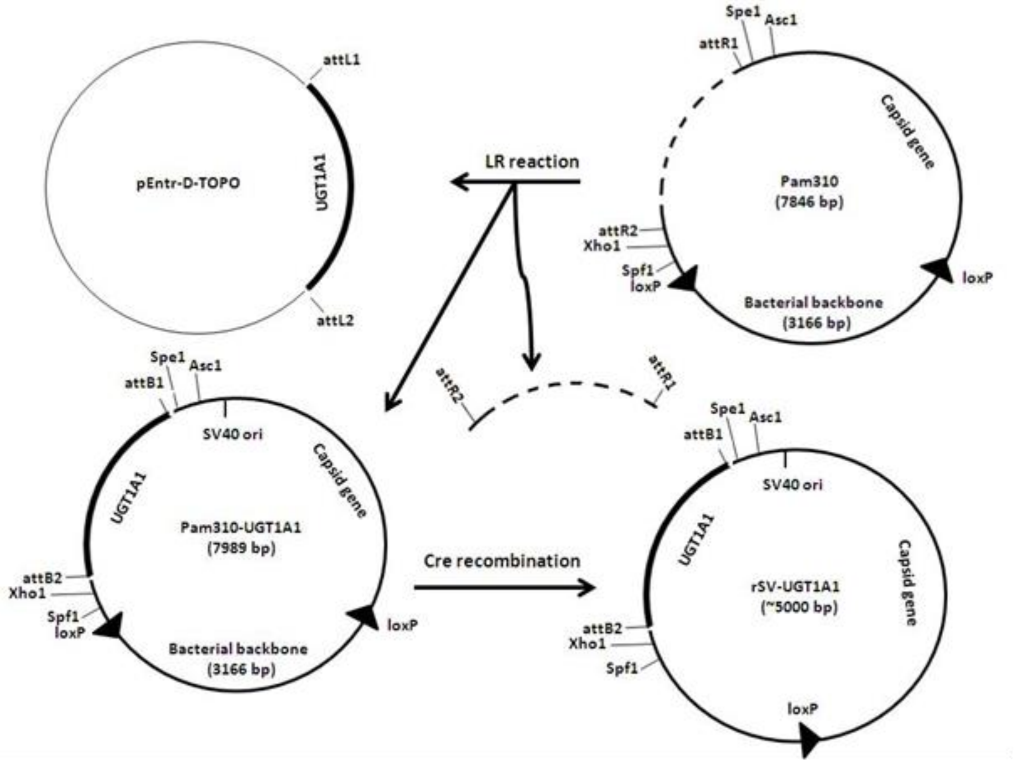

Figure S1. Construction of rSV-*hUGT1A1* vector. The human hUGT1A1 coding region was amplified, cloned to pEntr-d-TOPO vector and sequenced to exclude mutations. The resulting plasmid was used as the donor vector to insert the hUGT1A1 gene in the pAM310 destination vector by LR reaction[1].

Figure S2

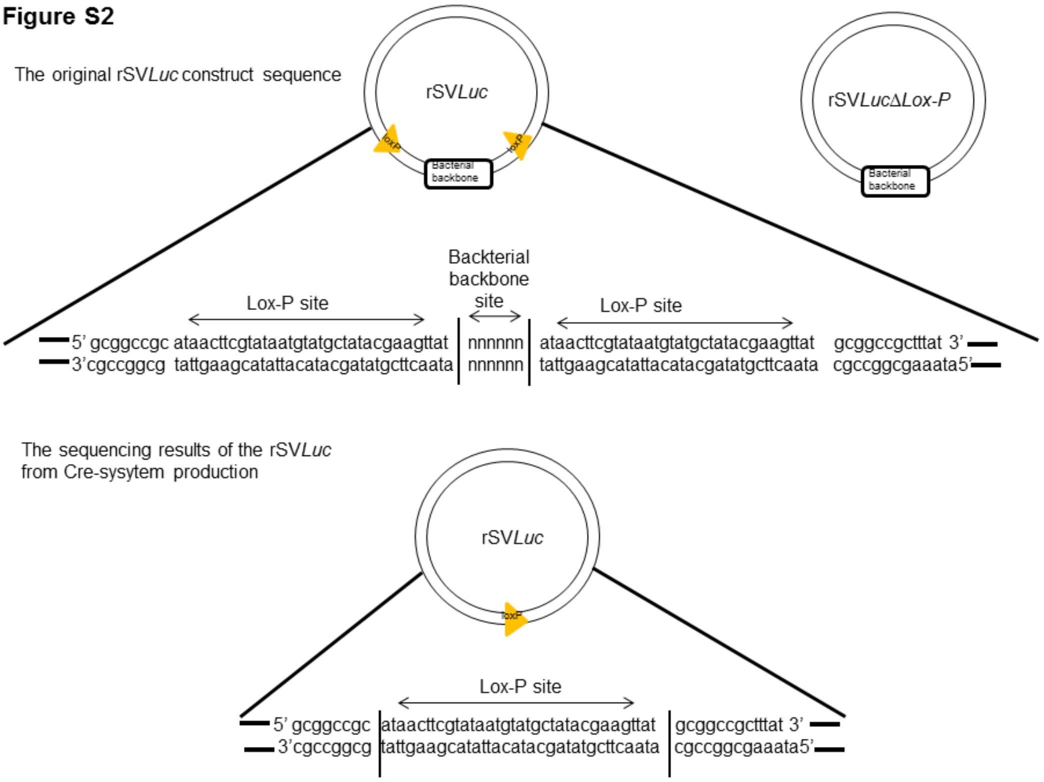

Figure S2. PCR amplification to confirm Cre mediated removal of the bacterial backbone from rSV*Luc*. For rSV-*hUGT1A1* and rSV*GFP* the same approach was used.

Figure S3

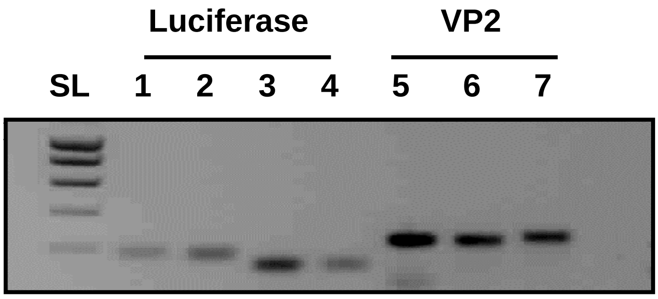

Figure S3. DIG-labeled PCR products. *Lane 1*, luciferase_1; *Lane 2*, luciferase_2; *Lane 3*, luciferase_3; *Lane 4*, luciferase_4; *Lane 5*, VP2_1; *Lane 6*, VP2_2; *Lane 7*, VP2_3.

Table S1

**Ampicillin standard curve**		**Virus**	
**log number**	**Ct value**	**batches**	**Ct value**
**0**	**32,018**	**rSV*Luc1***	**32,23839**
**1**	**29,307**	**rSV*Luc2***	**34,20386**
**2**	**26,289**	**rSV*Luc3***	**32,77787**
**3**	**23,722**	**rSV*Luc4***	**32,61454**
**4**	**20,092**		
**5**	**17,527**		
**6**	**15,348**		

Supplemental reference

1. Toscano, MG, van der Velden, J, van der Werf, S, Odijk, M, Roque, A, Camacho-Garcia, RJ, *et al.* (2017). Generation of a Vero-based packaging cell line for the production of SV40 gene delivery vector particles for use in clinical gene therapy studies. *Molecular Therapy-Methods & Clinical Development*.
