# Supplementary material for: Cre Recombinase Mediates the Removal of Bacterial Backbone to Efficiently Generate rSV40
Source: Mol Ther Methods Clin Dev. 2018 Feb 27;9:225–33. doi: 10.1016/j.omtm.2018.02.010 (PMC5948228; doi:10.1016/j.omtm.2018.02.010)
Supplement: Document S2. Article plus Supplemental Information [file mmc2.pdf]

Molecular Therapy
Methods & Clinical Development
Original Article

Cre Recombinase Mediates the Removal of Bacterial Backbone to Efficiently Generate rSV40

Xiaoxia Shi,[1] Matthew Ryan Ykema,[1] Jaco Hazenoot,[1] Lysbeth ten Bloemendaal,[1] Irene Mancini,[2] Machteld Odijk,[2] Peter de Haan,[2] and Piter J. Bosma[1]

[1]Tytgat Institute for Liver and Intestinal Research, Academic Medical Center, Amsterdam, the Netherlands; [2]Amarna Therapeutics B.V., Leiden, the Netherlands

Gene therapy has been shown to be a feasible approach to treat inherited disorders *in vivo*. Among the currently used viral vector systems, adeno-associated virus (AAV) vectors are the most advanced and have been applied in patients successfully. An important drawback of non-integrating AAV vectors is their loss of expression upon cell division, while repeating systemic administration lacks efficacy due to the induction of neutralizing antibodies. In addition, a significant percentage of the general population is not eligible for AAV-mediated gene therapy due to pre-existing immunity. Development of additional viral vectors may overcome this hurdle. Simian virus 40 (SV40)-derived vectors have been reported to transduce different tissues, including the liver, and prevalence of neutralizing antibodies in the general population is very low. This renders recombinant SV40 (rSV40) vector an interesting candidate for effective (re-)administration. Clinical use of SV40 vectors is in part hampered by less advanced production methods compared to AAVs. To optimize the production of rSV40 and make it better suitable for clinical practice, we developed a production system that relies on Cre recombinase-mediated removal of the bacterial plasmid backbone.

INTRODUCTION

Recent clinical successes demonstrate that gene therapy is a novel and effective treatment option for inherited genetic disorders.[1–4] The choice of gene therapy vector depends on the tissue and disease targeted. For rapidly dividing tissues, integrating vectors, i.e., lentiviral vectors, are essential to provide long-term correction.[5] For *in vivo* treatment of quiescent tissues, like the retina and the liver, non-integrating adeno-associated virus (AAV) vectors have been shown to provide long-term correction in pre-clinical animal models and in patients.[6,7] However, the efficacy of AAV-mediated liver-directed gene therapy is lost rapidly in young animals due to continuous hepatocyte replication in the growing liver.[8,9] Loss of efficacy is a hurdle toward this approach, since repeated treatment is not possible due to the host's immune responses toward the AAV vector induced by the first treatment.[10–13] Presently, AAV-mediated liver-directed gene therapy, therefore, is not applicable in young children. In addition, natural exposure to AAV is frequently early in life, and it results in the prevalence of neutralizing antibodies toward AAV in a significant percentage of the general population.[14] This renders gene therapy treatment with AAV in a part of the patients suffering from an inherited liver disease ineffective. Developing therapeutic strategies to overcome this problem will allow treatment of these patients and also of patients who have received a nontherapeutic vector dose in early phase studies.

Simian virus 40 (SV40)-based vectors could be a promising alternative to AAV vectors for gene replacement therapy for liver diseases. This virus has been widely studied as a gene delivery vector, and it was the first eukaryotic virus of which the entire genome was sequenced.[15,16] SV40 is a non-enveloped polyomavirus, containing a circular double-stranded DNA genome of 5,234 bp.[16,17] The SV40 early promoter drives the expression of the early gene encoding the large and small T antigens (LTag and STag). LTag is essential for genome replication and activation of the late promoter that drives the expression of the late gene encoding the capsid proteins VP1, VP2, and VP3.[18] To generate replication-defective SV40 vectors, the early gene is removed, leaving 2.7 kb of available space to clone exogenous genomic material. Absence of the early protein, the major SV40 antigen, prevents the production of all viral proteins.[19,20] Such replication-defective SV40 vectors are presumed to be non-immunogenic in humans, and systemic injection of SV40 vector particles does not elicit a detectable neutralizing antibody response, allowing repeated treatments.[21] Furthermore, the pre-existing immunity toward SV40 in the general population is very low.[22,23] In view of the limitations of the currently used vectors, the low prevalence of pre-existing immunity, and the possibility to re-inject make SV40 a highly appealing vector for treating mono-genetic disorders.

Other properties that render SV40 promising as a gene delivery vector are as follows: (1) its wide host range: SV40 enters the cell by binding the major histocompatibility complex class 1 (MHC1) present on the

Received 19 September 2017; accepted 22 February 2018;
https://doi.org/10.1016/j.omtm.2018.02.010.

Correspondence: Piter Bosma, Tytgat Institute for Liver and Intestinal Research, Academic Medical Center, S1-166, Meibergdreef 69, Amsterdam 1105 BK, the Netherlands
E-mail: p.j.bosma@amc.uva.nl

This is an open access article under the CC BY license (http://creativecommons.org/licenses/by/4.0/).

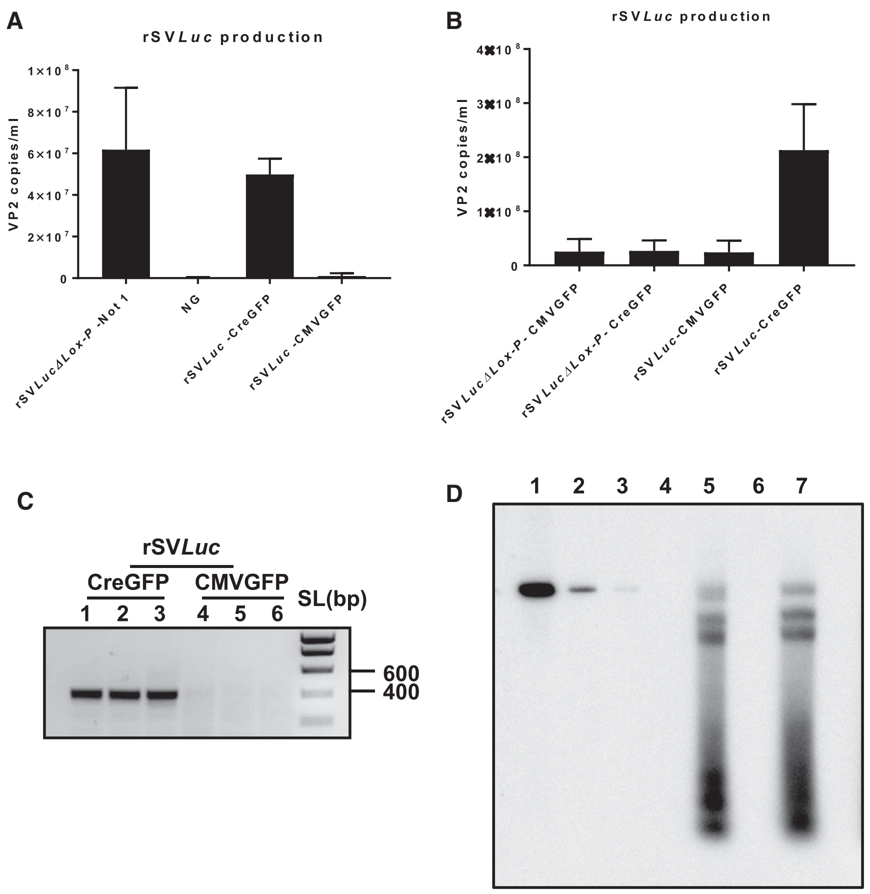

Figure 1. Cre-Mediated Production of rSV*Luc* Vector

(A) COS-1 cells were transfected with Not1-cut and re-circularized rSV*LucΔLox-P* or rSV*Luc* plasmid co-transfected with the Cre-expressing plasmid (CMV-Cre) or a control plasmid (CMV-GFP) in a 2:1 ratio. At 3–5 days after transfection, medium was analyzed for the presence of rSV*Luc* genomes using qPCR. Data represent the mean ± SD of triplicate experiments. (B) COS-1 cells were transfected with 2 μg rSV*Luc* or 2 μg rSV*LucΔLox-P* with Cre-GFP or CMV-GFP in a 2:1 ratio; 5 days later the presence of rSV*Luc* genomes was determined using qPCR. Data represent the mean ± SD of nine repeat experiments. (C) The medium of COS-1 cells co-transfected of rSV*Luc* with CMV-Cre or CMV-GFP plasmid was used to confirm the removal of the bacterial backbone using PCR. (D) Southern blot of rSVLuc vectors. Lanes 1–3, 10^8, 10^7, and 10^6 copies of Not1-linearized rSV*Luc* plasmid, respectively; lanes 4 and 6, empty; lane 5, 10^{10} copies vector genomes of an rSV*Luc* batch produced using Not1-mediated excision of the rSV*Luc* vector genome; lane 7, 10^9 copies of an rSV*Luc* batch produced by Cre recombinase-mediated excision of the rSV40 vector genome. In rSV*LucΔLox-P*, the viral genome was not flanked by Lox-P sites. NG, no transfection.

membrane of most cell types;[24–28] (2) transduction of different tissues, such as liver, spleen, kidney, and bone marrow, with gene transfer percentages as high as 80%–90% having been reported;[29–31] and (3) long-term transgene expression: when transgene expression is achieved, the level is generally consistent in experiments lasting well over 1 year *in vivo* and *in vitro* (random integration could play a role in prolonged expression, albeit lower than the levels obtained with AAV vectors).[29–31]

Despite these promising characteristics, the use of SV40 vectors to correct inherited diseases in pre-clinical animal models is very limited, which in part is due to the lack of an efficient production method. One of the steps complicating the production of rSV40 is the need to excise the vector genome from the bacterial backbone. This step is needed because of the size restraints of the SV40 capsid.[29] In addition, this removal is needed for reasons of safety, as the presence of the *E. coli* origin and an antibiotic resistance gene is unwanted. Currently, removal of the bacterial backbone is done by restriction enzyme cutting, gel purification, and self-ligation to generate the circular SV40 vector genome that can be transfected into a packaging cell line, such as COS-1 or COS-7, and propagated.[32–35] This method, however, is time consuming, expensive, and results in lower transfection efficiencies due to the relaxed DNA conformation of the re-circularized plasmids. To overcome this, we tested the feasibility of producing SV40 vector particles using Cre recombinase-mediated removal of the bacterial backbone. To this end, an SV40 vector destination plasmid pSV*ac* was used, in which the vector genome is flanked by Lox-P sites to allow specific removal of the bacterial backbone by Cre recombinase expressed in SV40 vector packaging cells.[36] We here show that expression of Cre recombinase in COS-1 and SuperVero cells results in an efficient removal of the bacterial backbone.

RESULTS

Cre Recombinase-Mediated Production of rSV*Luc*

COS-1 cells were transfected with the following: (1) the rSV*Luc*[36] plasmid with Lox-P sites flanking the rSV genome; (2) the rSV*LucΔLox-P* plasmid without Lox-P site co-transfected with a Cre-expressing plasmid; and, (3) as a negative control, a GFP-expressing plasmid. At 5 days after transfection, the medium was harvested and the titer of rSV*Luc* was determined using qPCR. The number of vector genomes produced by the Cre-mediated excision of the SV40 genome from rSV*Luc* co-transfected with the Cre plasmid was comparable to that produced by the Not1-mediated excision of the rSV40 genome from the rSV*LucΔLox-P* plasmid (Figure 1A). Co-transfection with a CMV-GFP plasmid, not expressing Cre, did not result in a significant production of rSV40 (Figure 1B). The Cre-mediated excision of the rSV40 genome was confirmed by amplification with primers flanking the Lox-P sites, resulting in an amplicon of the expected size only upon co-transfection of rSV*Luc* with the Cre-expressing plasmid (Figure 1C). To further confirm Cre-mediated removal, the amplicons were sequenced showing a single Lox-P site flanked by

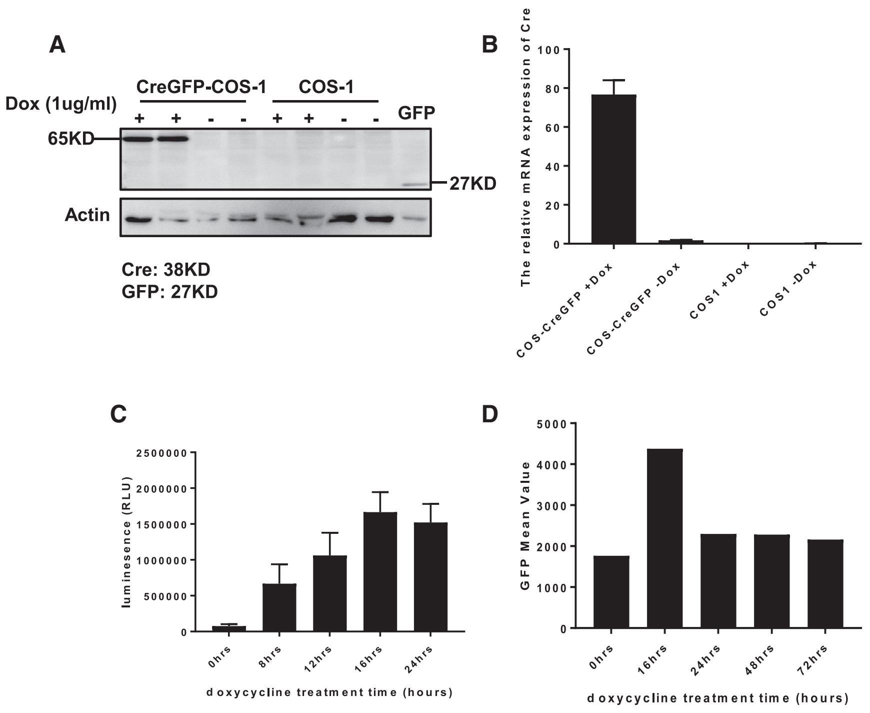

Figure 2. Production of rSV40 by COS-1 Cells with Inducible Cre Expression

(A and B) COS-1 cells were transduced with a lentiviral vector with inducible Cre expression and selected by neomycin. Selected cells (CreGFP-COS-1) and parental COS-1 cells were treated with 1 μg/mL doxycycline for 48 hr. The presence of Cre protein was determined by western blotting (A) and of Cre mRNA by qRT-PCR (B). (C and D) CreGFP-COS-1 cells were incubated with 1 μg/mL doxycycline for 0, 8, 12, 16, 24, 48, and 72 hr before transfection with rSV*Luc* or rSV*GFP* plasmids. Medium containing rSV*Luc* or rSV*GFP* was collected 3 days after transfection and used to infect COS-1 cells, followed by measuring the luciferase expression by luminescence (C) and determining the GFP expression by FACS (D). Data represent the mean ± SD of triplicate experiments.

the expected viral genome sequences, thereby indeed demonstrating Cre-mediated recombination (Figure S2). Furthermore, the Southern blot analysis results showed that the vector genomes present in rSV40 preparations produced by both methods are very similar (Figure 1D). Furthermore, removal of the bacterial backbone was tested by performing a qPCR for the ampicillin gene. Using this highly sensitive method, with a lower detection limit <10 copies, in all rSV*Luc* batches tested, no signal for ampicillin could be detected (Table S1).

Inducible Cre-Expressing Packaging COS-1 Cell-Mediated Production of rSV40

To further optimize the rSV40 production method, we generated a stable COS-1 cell line with doxycycline-inducible expression of Cre-GFP. Upon the addition of doxycycline, a strong increase of Cre recombinase protein and mRNA was seen (Figures 2A and 2B). To determine the optimal time of Cre induction for the efficient production of rSV40, rSV*Luc* and rSV*GFP* plasmids were transfected to the cells at different time points after doxycycline addition. The rSV40-containing medium was harvested at 3 days after transfection and used to infect COS-1 cells. The luciferase expression (Figure 2C) and GFP expression, detected by fluorescence-activated cell sorting (FACS) (Figure 2D), both showed that 16 hr of Cre induction prior to the transfection is sufficient for efficient rSV40 production. After 16 hr the efficiency of viral production did not increase further (Figures 2C and 2D). Thus, 16 hr of doxycycline pre-treatment is sufficient to use the Cre-inducible cell line to produce rSV40.

The significant difference in viral genome titer of doxycycline-treated and untreated cells clearly demonstrates that Cre expression is needed for efficient rSV40 vector production (Figure 3A). To further confirm Cre-mediated recombination and excision of the rSV40 genome, the Lox-P-flanking region was amplified. Amplicons with the expected size were present in the medium from cells both treated and untreated with doxycycline (Figure 3B), as subsequently confirmed by sequence analysis (sequencing data not shown). A comparison between this Cre-expressing cell line and co-transfection of an rSV40 construct with a Cre-expressing plasmid showed that both methods resulted in a comparable titer of rSV40 (Figure 3C).

COS-1-indCre-Mediated Production of rSV-*hUGT1A1*

To demonstrate the efficacy of the novel rSV40 production method for a therapeutic application of rSV40, the human UGT1A1 cDNA was inserted into pAM310. A UGT1A1-encoding vector can be used to correct UGT1A1 deficiency, as seen in Crigler-Najjar syndrome. An rSV-*hUGT1A1* plasmid was transfected to Cre-GFP-COS-1 cells, which were pre-treated with doxycycline for 16 hr. At 3 days after transfection, the medium was harvested and the titer of rSV-h*UGT1A1* was determined by qPCR. The number of vector genomes in the medium, assayed by qPCR, demonstrated that rSV40 particles are formed and released into the medium upon the induction of Cre expression (Figure 4A). Next, PCR (Figure 4B) and sequencing confirmed Cre-mediated excision of the rSV40 genome. To demonstrate the functionality of the produced rSV-*hUGT1A1*, the viral vector was used to transduce COS-1 cells. The expression of hUGT1A1 in the transduced cells confirmed that functional rSV40-*hUGT1A1* vectors were produced (Figure 4C).

SuperVero-indCre-Mediated Production of rSV40s

To generate a cell line for efficient production of clinical grade rSV40s, we generated SuperVero cells with inducible Cre expression (Figures 5A and 5B), and we used these cells to produce rSV*Luc* and rSV-*hUGT1A1*. The results showed that inducible Cre-expressing SuperVero cells can produce rSV40 vector without contamination

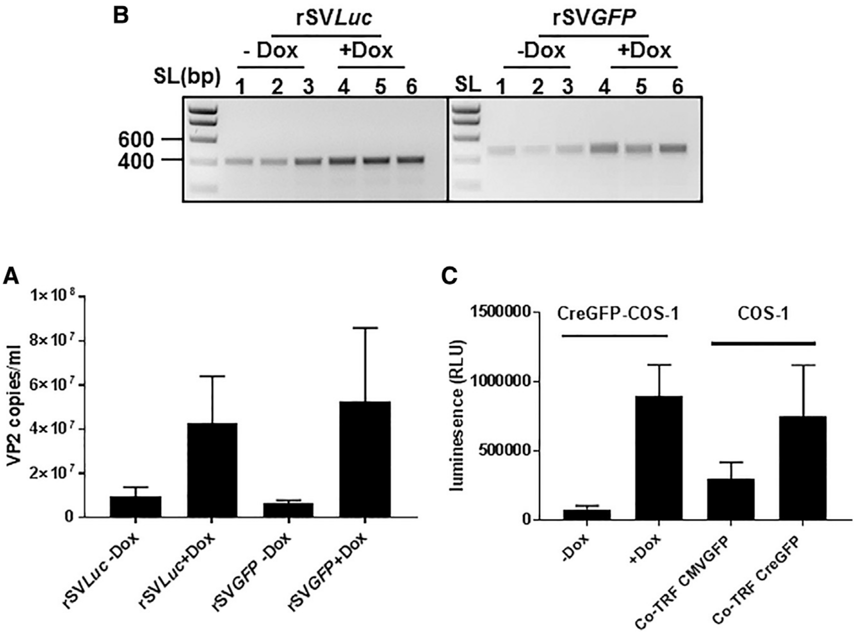

Figure 3. Efficient rSV40 Production by Cre-GFP COS-1 Cells

(A) Cre-GFP COS-1 cells were incubated with doxycycline (1 μg/mL), after 16 hr transfected with rSV*Luc* or rSV*GFP* plasmid, and 3 days later the titers in the medium were determined as measured by qPCR. (B) PCR amplification of the Lox-P site remaining in the rSV*Luc* and rSV*GFP* vectors present in the medium. (C) The efficiency of rSV40 production by COS-1 co-transfected with rSV*Luc* and a Cre-expressing plasmid and by Cre-GFP COS-1 transfected with rSV*Luc* plasmid only. The rSV*Luc* vectors produced were used to transduce COS-1 cells, and luciferase expression was measured 3 days later. Data represent the mean ± SD of triplicate experiments.

of detectable levels of replication-competent Tag-positive wild-type (WT) SV40 (the large T antigen was undetectable using qPCR). The number of vector genomes in the medium assayed by qPCR demonstrated that rSV40 particles are formed and released into the medium upon the induction of Cre expression (Figure 5C), and PCR (Figure 5D) confirmed Cre-mediated excision of the rSV40 genome. The luminescence intensity (Figure 5E) and western blot results (Figures 5F and 5G) demonstrated the functionality of the produced rSV*Luc* and rSV-*hUGT1A1*, respectively.

DISCUSSION

Many papers have reported the use of rSV40 vectors to treat inherited disorders in pre-clinical animal models.[30,37,38] The wide host range renders it a promising vector not only for the liver but also for organs like the brain and the lung using local administration.[39,40] Upon systemic injection, rSV40 mainly targets the hepatocytes, and the dispersion to other tissues can be reduced further by administration into the hepatic artery or portal vein.[41] Although SV40 gives good expression in human cell lines *in vivo* in mice and rat, the expression levels provided by rSV40 are clearly lower compared to AAV. The cause of this lower expression levels *in vivo* is unclear but could be species specific, for instance, due to a low affinity of the murine MHC1 for SV40, preventing efficient cell entry.

All *in vivo* studies used restriction enzyme-mediated excision of the viral genome from the plasmid, a time consuming, expensive, and laborious method, requiring agarose gel purification and overnight ligation. To optimize rSV40 production, two Lox-P sites flanking the SV40 genome have been inserted to allow Cre-mediated excision of the genome from the bacterial backbone. When compared to the restriction enzyme-mediated removal, the Cre-mediated excision by co-transfecting a Cre-expressing plasmid was at least comparable. For the Not1-mediated excision, more plasmid was required to compensate for the lower transfection efficiency of the relaxed plasmid structure, resulting from the enzyme-mediated removal followed by overnight ligation compared to the super-coiled plasmid structure used with co-transfection.[42] Thus, the efficiency of rSV40 production using co-transfection of a Cre-expressing plasmid with the rSV40 plasmid appears comparable to that of the Not1-mediated excision. In addition, the absence of a signal for ampicillin in a sensitive qPCR demonstrates that Cre-mediated excision of the rSV40 vector genome results in effective removal of the bacterial backbone from the produced rSV40 batches. Furthermore, a Southern blot of rSV*Luc* produced using Not1 alongside Cre-mediated excision revealed a very similar pattern, further confirming both methods result in the production of similar rSV40 batches. The smear seen in both preparations may result from incomplete DNase digestion of non-packaged genomes or from small pieces of SV40 genomes packaged upon prolonged production.[43] The presence of these partial SV40 genomic fragments could also explain the background signal that, albeit very low, is detectable by qPCR in the absence of Cre expression (Figure 1A). Sequencing of the Lox-P site-containing region demonstrated that the rSV40 genome was indeed excised by Cre-mediated recombination.

To further improve and standardize the production method for rSV40, we subsequently generated production cell lines with inducible expression of Cre recombinase. The COS-1 cell, the monkey kidney cell line expressing the rSV40 Tag and capsid genes, is used for the production of rSV40 by most groups.[31,32,44] Upon transduction with pInd20-Cre-GFP, and neomycin selection, a COS-1 cell line with inducible expression of Cre was generated. A 16-hr treatment with doxycycline to induce the expression of Cre appeared optimal for rSV40 production. Longer incubation times with doxycycline resulted in a lower production efficiency. Apparently, the prolonged presence of high levels of Cre impairs rSV40 replication and/or production. The most likely explanation for this is that prolonged expression of Cre could cause recombination

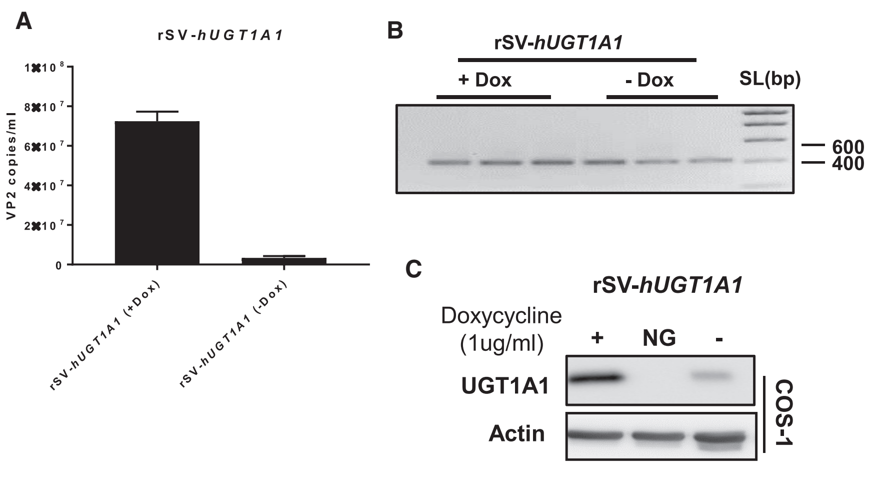

Figure 4. Efficient Production of rSV40 with a Therapeutic Gene by Cre-GFP COS-1 Cells

(A) Cre-GFP COS-1 cells were incubated with doxycycline (1 μg/mL) for 16 hr followed by transfection with the rSV-*hUGT1A1* plasmid. 3 days later the vector titer was determined by qPCR. Data represent the mean ± SD of six repeat experiments. (B) PCR amplification of the Lox-P sites. (C) COS-1 cells were transduced with rSV-*hUGT1A1*, and 3 days thereafter UGT1A1 expression was determined using western blotting. NG, negative control, means no transduction.

between two rSV40 vector genomes, resulting in an unwanted product too large to be packaged.[45] In the absence of doxycycline, the low levels of rSV40 genome could be detected. This is most likely due to low-level expression of Cre due to leakiness of the promoter. The qPCR data show that, in the absence of doxycycline, Cre expression, albeit very low, is seen, confirming the data reported by others.[46–48] Upon the addition of doxycycline, the production of rSV40 is much higher, showing that Cre expression is limited in the absence of doxycycline. The efficiency of the production was similar to that obtained after co-transfecting the rSV40 plasmids with the Cre-expressing plasmid. Comparison with the Not1-mediated excision of viral genome is difficult since the amounts of plasmid used for that method are higher, but both Cre-based methods have comparable efficiency.

By using the inducible Cre-expressing COS-1 cells, we produced rSV40 viral vectors encoding reporter genes; the firefly luciferase; *GFP*; and a therapeutic gene, *UGT1A1*. Because Tag has been removed from the rSV40 plasmids, it has to be provided in *trans* by the production cell line, such as COS-1. This cell line contains an integrated copy of the SV40, mutated at its origin of replication, in its genome, and it is used by most groups to produce rSV40.[44,49–51] A major drawback of this cell line is that, due to extensive overlap between the integrated genome and the rSV40 plasmids, homologous recombination is possible. As a result, there is a risk of generation replication-competent wild-type SV40 revertants when producing rSV40 in this cell line. In view of the tumorigenic potential of the LTag proteins, the presence of WT SV40 contaminants in clinical grade batches of rSV40 vector needs to be avoided. In our results, LTag containing WT SV40 in rSV40 vector produced by COS-1 during the first three passages was undetectable, but it was detected thereafter. The generation of LTag-positive SV40 over time is similar to that reported by other groups that could also detect WT SV40 after the fourth amplification round.[43,49] The chance of homologous recombination to occur can be minimized by reducing the sequence overlap between the plasmid and the SV40 genes integrated in the production cell line.

Recently, a novel Vero-based packaging cell line to produce rSV40 called SuperVero was generated.[36] In this novel packaging cell line, only the LTag gene is integrated into the chromosomal DNA. Because sequence overlap between chromosomally inserted SV40 LTag-coding sequences and episomally replicating SV40 vector sequences is lacking, the emergence of replication-competent particles during the vector production process is highly unlikely.[36] To further improve the production of rSV40, we therefore generated a stable SuperVero cell line with inducible Cre expression to be used for the production of rSV40 vectors. Our data show that efficient production of rSV40 is possible with this cell line upon the induction of Cre expression using doxycycline (Figure 5C). The promoter used appeared to be a bit leaky since some expression of Cre was detectable in the absence of doxycycline. This could explain the production of rSV40 by these cells, albeit much less efficient, without doxycycline induction.

In conclusion, by inserting Lox-P sites in the bacterial backbone of rSV40 plasmids, we have developed an efficient rSV40 production method, which is more convenient and time saving than other methods for producing SV40 vectors. This method can be used in our novel SuperVero cell line, allowing an efficient production of clinical-grade rSV40 vectors.

MATERIALS AND METHODS

Generation of rSV-*hUGT1A1*

The primers hUGT1A1F (5’-CACCGCCCTGTCTCCTCAGCTTCA-3’) and hUGT1A1R (5’-TCAATGGGTCTTGGATTTGTG-3’) were used to amplify the hUGT1A1 cDNA for gateway-mediated entry into pENTR/D/TOPO (Invitrogen). After sequencing to check for mutations, the hUGT1A1 was inserted into the attR1 and attR2 sites of the destination vector pAM310 (AMARNA, Leiden) by LR recombinase, resulting in rSV-*hUGT1A1*.

Production of rSV40

Not1-mediated excision of the rSV40 genome from the plasmids was performed as reported in Kondo et al.[21] In short, 20 μg plasmid was cut, and the rSV40 genome band was isolated from a 0.7% agarose gel using Zippy Clean (Zymoresearch, Freiburg, Germany) and ligated in an overnight ligation using 10 units/μg DNA T4

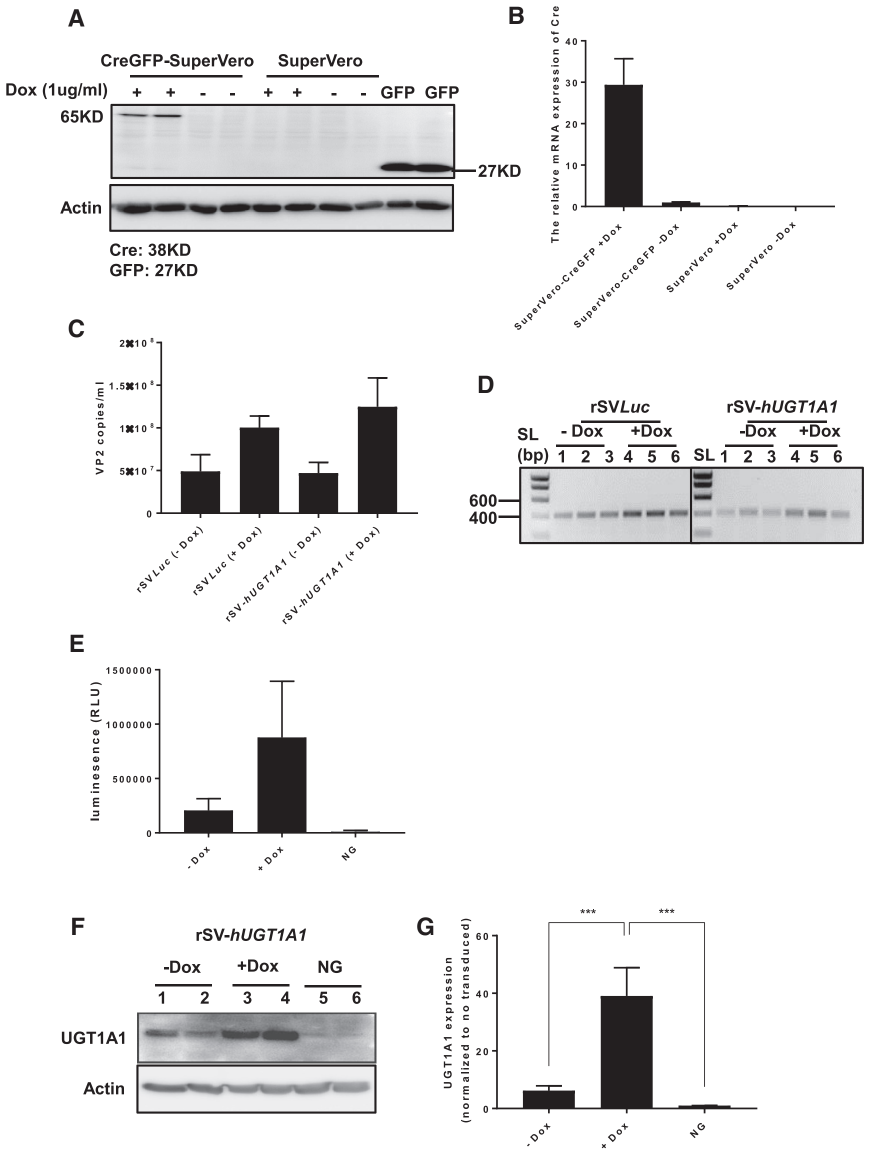

Figure 5. Efficient Production of rSV40 Vectors by Cre-GFP SuperVero Cells

SuperVero cells were transduced with an inducible Cre-expressing lentiviral vector and selected using neomycin. (A and B) The resulting Cre-GFP SuperVero cells and the parental SuperVero cells were treated with 1 μg/mL doxycycline for 48 hr, and the levels of Cre protein (A) and mRNA (B) were determined. (C) Cre induction stimulates the production of rSV40 vectors by Cre-GFP SuperVero cells. Data represent the mean ± SD of triplicate experiments. (D) PCR amplification of the Lox-P sites of rSV*Luc1* and rSV-*hUGT1A1* vectors produced by Cre-GFP SuperVero cells. (E) Luciferase and (F and G) UGT1A1 expression in SuperVero cells transduced by rSVLuc and rSVhUGT1A1 (two-tailed Student's t test, ***p <0.001). Data represent the mean ± SD of six repeat experiments. No TRD, no transduction.

DNA ligase (Promega, Benelux, Leiden). The re-circularized rSV40 genome was transfected into COS-1 or SuperVero cells using Polyethylenimine (PEI). At 3 days after the transfection, the medium was collected, filtered through a 0.45-μm filter, aliquoted, and stored at −80°C.

Cre recombinase excision of the bacterial backbone was performed by co-transfecting rSV40 plasmids with a CMV-Cre plasmid, in a ratio of 2: 1 using PEI into COS-1 cells, with a CMV-GFP plasmid as a negative control. The medium from the transfected COS-1 cells was collected at day 5 after transfection, filtered through a 0.45-μm filter, aliquoted, and stored at −80°C.

These seeding stocks were used to infect fresh COS-1 cells using 400 vector genomes/cell, and medium was collected at day 3, filtered, aliquoted, and stored at −80°C. The titer of these vector batches varied between 10^7 and 10^8 vg/mL.

Southern Blot Hybridization of rSV40

Vector DNA from rSV*Luc* batches was purified and isolated using phenol-chloroform extraction. The luciferase and SV40 capsid gene VP2 primers (see Table 1) were designed for making DIG-labeled probe by PCR using the Roche DIG-probe synthesis kit (Roche, Germany). As a positive control, rSV*Luc* plasmid, was linearized by Not1 digestion, and a different number of DNA copies was loaded alongside the purified rSV*Luc* vector DNA on a 0.8% agarose gel. After electrophoresis, the DNA was blotted to a Hybond XL membrane overnight, hybridized with luciferase and VP2 DIG-labeled probe, and visualized by LAS4000.

qPCR Titer Determination of rSV40

Samples were treated with 1 μL/10 μL medium DNase (1 unit/μL) (Promega, Benelux, Leiden) for 30 min at 37°C. DNase was inactivated at 95°C for 5 min. Primers toward firefly luciferase, GFP, luciferase, UGT1A1, or the SV40 capsid gene VP2 were used to perform the qPCR. Standard curves were generated using 10-fold serial dilutions of the rSV40 production plasmids. The qPCR was done using Fast SYBR Green Master Mix (Thermo Fisher Scientific, CA, USA) in an LC480 (Light Cycler; Roche) at 95°C for 5 min, followed by 45 cycles at 95°C for 10 s, 60°C for 10 s, and 72°C for 15 s.

Confirming Cre Recombinase Excision of the rSV40 Vector Genome

rSV40-containing medium was treated with DNase (1 μL/10 μL medium DNase [1 unit/μL]) (Promega, Benelux, Leiden) for 30 min at 37°C. DNase was inactivated at 95°C for 5 min. A region encompassing the Lox-P site was amplified using a primer in the

Table 1. Primers Used to Make DIG-Labeled Probe

Primer	Sequence (5'-3')	Product (bp)
Luciferase_1_forward	ACACCCGAGGGGGATGATAA	126
Luciferase_1_reverse	TCTCACACACAGTTCGCCTC	
Luciferase_2_forward	ACTGGGACGAAGACGAACAC	112
Luciferase_2_reverse	GGGTGTTGGAGCAAGATGGA	
Luciferase_3_forward	TGGGCGCGTTATTTATCGGA	90
Luciferase_3_reverse	GCTGCGAAATGCCCATACTG	
Luciferase_4_forward	TTTGGAGCACGGAAAGACGA	89
Luciferase_4_reverse	CTCCTCCGCGCAACTTTTTC	
VP2_1_forward	TTGGTGGGGAACCTTTGGAG	175
VP2_1_reverse	GGATCAGGAACCCAGCACTC	
VP2_2_forward	TGCTGTTGACATTTGTGGGC	180
VP2_2_reverse	ATCATAGGCTGCCCATCCAC	
VP2_3_forward	CCTAGGCTCACCTCACAGGA	196
VP2_3_reverse	ACCCTTCCCTGTTGGCTACT	

VP1 sequence and a primer in the luciferase, GFP, or hUGT1A1 gene sequence, respectively, followed by sequencing of the resulting amplicons. Primers used for the different constructs are listed in Table 2.

Generating Producer Cell Lines with Inducible Cre Expression

For the preparation of a Cre-expressing lentiviral vector, using LR recombinase, a GFP-tagged Cre gene was introduced to pInducer20[52] (Addgene) vector. The resulting pInducer-Cre-GFP plasmid was co-transfected with pMDLg, pRSV-REV, and pVSVg to HEK293T cells using PEI to produce lentiviral vector, as reported previously.[53] These lentiviral vectors were used to transduce COS-1 or SuperVero cells followed by neomycin selection to generate a stable production cell line with inducible Cre expression.

Producing rSV40 Vectors Using Inducible CreGFP-COS-1 or CreGFP-SuperVero Cells

COS-1 or SuperVero cells were incubated with 1 μg/mL doxycycline for different times before the PEI-mediated transfection of rSV40 plasmids was performed. rSV40 particles were harvested from the medium 3 days after transfection, and the titer of the rSV40 vectors produced was determined by qPCR using primers toward the VP2 gene.

rSV*Luc* and rSV*GFP* Transduction

COS-1 cells were transduced with 100 μL rSV*Luc* with 400 vector genomes/cell in a 96-well plate. At 3 days after transduction, the luminescence intensity of transduced cells was measured by ELISA reader using ONE-Glo Luciferase Assay System (Promega, Benelux, Leiden). COS-1 cells were transduced with rSV*GFP* with 400 vector genomes/cell in a 12-well plate. At 3 days after transduction, the cells were harvested and GFP mean fluorescence was measured using flow cytometry.

Table 2. Primers Used to Amplify the Lox-P-Containing Region

Primer	Sequence (5'-3')
VP2	TGGGCAGCCTATGATTGGAA
Luciferase	GCGGAAAGATCGCCGTGTAA
GFP	TTCGTAGAGCAGCACGAGAC
hUGT1A1	AGCCCACAAATCCAAGACCC

rSV-*hUGT1A1* Transduction

COS-1 cells were transduced in 6-well plates with 400 vector genomes/cell of rSV-*hUGT1A1*; 48 hr after transduction, the cells were washed once with PBS and cells were lysed with radioimmunoprecipitation assay (RIPA; 50 mM Tris [pH 8.0], 150 mM NaCl, 1% Triton X-100, 0.5% Na-deoxycholate, and 0.1% SDS) buffer and Protease Inhibitor (1:100 dilution) (Roche, Germany) for 20 min. 30 μg cell lysate was loaded onto a 10% Acrylamide gel and blotted to polyvinylidene fluoride (PVDF) membrane (semi-dry blotting, 1 hr, 0.05 mA per gel). A monoclonal antibody toward UGT1A1 (1:700 dilution) followed by a goat anti-mouse HRP-labeled second antibody (Dakoplast, the Netherlands) (1:5,000) was used to detect expression of UGT1A1, as described previously.[54]

Statistics

All results are given as mean ± SD. Statistical significance was determined by two-tailed Student's t test using GraphPad Prism 7 (GraphPad, La Jolla, CA, USA).

SUPPLEMENTAL INFORMATION

Supplemental Information includes three figures and one table and can be found with this article online at https://doi.org/10.1016/j.omtm.2018.02.010.

AUTHOR CONTRIBUTIONS

X.S. and P.J.B. designed and wrote the manuscript; M.R.Y., J.H., and L.t.B. contributed to all experiments, including generation of the constructs; I.M., M.O., and P.d.H. provided the SV*GFP*, SV*Luc* plasmids and the SuperVero cell line and critically read the manuscript.

ACKNOWLEDGMENTS

X.S. is the recipient of a fellowship from the China Scholarship Council (CSC). M.R.Y. received a Fulbright Scholarship from the U.S. State Department.

REFERENCES

1. Smith, B.K., Collins, S.W., Conlon, T.J., Mah, C.S., Lawson, L.A., Martin, A.D., Fuller, D.D., Cleaver, B.D., Clément, N., Phillips, D., et al. (2013). Phase I/II trial of adeno-associated virus-mediated alpha-glucosidase gene therapy to the diaphragm for chronic respiratory failure in Pompe disease: initial safety and ventilatory outcomes. Hum. Gene Ther. *24*, 630–640.
2. Herson, S., Hentati, F., Rigolet, A., Behin, A., Romero, N.B., Leturcq, F., Laforêt, P., Maisonobe, T., Amouri, R., Haddad, H., et al. (2012). A phase I trial of adeno-associated virus serotype 1-γ-sarcoglycan gene therapy for limb girdle muscular dystrophy type 2C. Brain *135*, 483–492.

3. Jacobson, S.G., Cideciyan, A.V., Ratnakaram, R., Heon, E., Schwartz, S.B., Roman, A.J., Peden, M.C., Aleman, T.S., Boye, S.L., Sumaroka, A., et al. (2012). Gene therapy for leber congenital amaurosis caused by RPE65 mutations: safety and efficacy in 15 children and adults followed up to 3 years. Arch. Ophthalmol. *130*, 9–24.

4. Blaese, R.M., Culver, K.W., Miller, A.D., Carter, C.S., Fleisher, T., Clerici, M., Shearer, G., Chang, L., Chiang, Y., Tolstoshev, P., et al. (1995). T lymphocyte-directed gene therapy for ADA- SCID: initial trial results after 4 years. Science *270*, 475–480.

5. Miyoshi, H., Smith, K.A., Mosier, D.E., Verma, I.M., and Torbett, B.E. (1999). Transduction of human CD34+ cells that mediate long-term engraftment of NOD/SCID mice by HIV vectors. Science *283*, 682–686.

6. Wang, L., Nichols, T.C., Read, M.S., Bellinger, D.A., and Verma, I.M. (2000). Sustained expression of therapeutic level of factor IX in hemophilia B dogs by AAV-mediated gene therapy in liver. Mol. Ther. *1*, 154–158.

7. Nathwani, A.C., Tuddenham, E.G., Rangarajan, S., Rosales, C., McIntosh, J., Linch, D.C., Chowdary, P., Riddell, A., Pie, A.J., Harrington, C., et al. (2011). Adenovirus-associated virus vector-mediated gene transfer in hemophilia B. N. Engl. J. Med. *365*, 2357–2365.

8. Paulk, N.K., Wursthorn, K., Wang, Z., Finegold, M.J., Kay, M.A., and Grompe, M. (2010). Adeno-associated virus gene repair corrects a mouse model of hereditary tyrosinemia in vivo. Hepatology *51*, 1200–1208.

9. Flageul, M., Aubert, D., Pichard, V., Nguyen, T.H., Nowrouzi, A., Schmidt, M., and Ferry, N. (2009). Transient expression of genes delivered to newborn rat liver using recombinant adeno-associated virus 2/8 vectors. J. Gene Med. *11*, 689–696.

10. Calcedo, R., and Wilson, J.M. (2013). Humoral immune response to AAV. Front. Immunol. *4*, 341.

11. Mingozzi, F., and High, K.A. (2013). Immune responses to AAV vectors: overcoming barriers to successful gene therapy. Blood *122*, 23–36.

12. Tse, L.V., Moller-Tank, S., and Asokan, A. (2015). Strategies to circumvent humoral immunity to adeno-associated viral vectors. Expert Opin. Biol. Ther. *15*, 845–855.

13. Harbison, C.E., Weichert, W.S., Gurda, B.L., Chiorini, J.A., Agbandje-McKenna, M., and Parrish, C.R. (2012). Examining the cross-reactivity and neutralization mechanisms of a panel of mAbs against adeno-associated virus serotypes 1 and 5. J. Gen. Virol. *93*, 347–355.

14. Boutin, S., Monteilhet, V., Veron, P., Leborgne, C., Benveniste, O., Montus, M.F., and Masurier, C. (2010). Prevalence of serum IgG and neutralizing factors against adeno-associated virus (AAV) types 1, 2, 5, 6, 8, and 9 in the healthy population: implications for gene therapy using AAV vectors. Hum. Gene Ther. *21*, 704–712.

15. Fiers, W., Contreras, R., Haegemann, G., Rogiers, R., Van de Voorde, A., Van Heuverswyn, H., Van Herreweghe, J., Volckaert, G., and Ysebaert, M. (1978). Complete nucleotide sequence of SV40 DNA. Nature *273*, 113–120.

16. Reddy, V.B., Thimmappaya, B., Dhar, R., Subramanian, K.N., Zain, B.S., Pan, J., Ghosh, P.K., Celma, M.L., and Weissman, S.M. (1978). The genome of simian virus 40. Science *200*, 494–502.

17. Fanning, E., and Knippers, R. (1992). Structure and function of simian virus 40 large tumor antigen. Annu. Rev. Biochem. *61*, 55–85.

18. Daniels, R., Sadowicz, D., and Hebert, D.N. (2007). A very late viral protein triggers the lytic release of SV40. PLoS Pathog. *3*, e98.

19. Ahuja, D., Sáenz-Robles, M.T., and Pipas, J.M. (2005). SV40 large T antigen targets multiple cellular pathways to elicit cellular transformation. Oncogene *24*, 7729–7745.

20. Zhu, J.Y., Rice, P.W., Chamberlain, M., and Cole, C.N. (1991). Mapping the transcriptional transactivation function of simian virus 40 large T antigen. J. Virol. *65*, 2778–2790.

21. Kondo, R., Feitelson, M.A., and Strayer, D.S. (1998). Use of SV40 to immunize against hepatitis B surface antigen: implications for the use of SV40 for gene transduction and its use as an immunizing agent. Gene Ther. *5*, 575–582.

22. Garcea, R.L., and Imperiale, M.J. (2003). Simian virus 40 infection of humans. J. Virol. *77*, 5039–5045.

23. Barbanti-Brodano, G., Sabbioni, S., Martini, F., Negrini, M., Corallini, A., and Tognon, M. (2004). Simian virus 40 infection in humans and association with human diseases: results and hypotheses. Virology *318*, 1–9.

24. Stang, E., Kartenbeck, J., and Parton, R.G. (1997). Major histocompatibility complex class I molecules mediate association of SV40 with caveolae. Mol. Biol. Cell *8*, 47–57.

25. Norkin, L.C. (1999). Simian virus 40 infection via MHC class I molecules and caveolae. Immunol. Rev. *168*, 13–22.

26. Anderson, H.A., Chen, Y., and Norkin, L.C. (1998). MHC class I molecules are enriched in caveolae but do not enter with simian virus 40. J. Gen. Virol. *79*, 1469–1477.

27. Bernacchi, S., Mueller, G., Langowski, J., and Waldeck, W. (2004). Characterization of simian virus 40 on its infectious entry pathway in cells using fluorescence correlation spectroscopy (Portland Press Limited).

28. Tsai, B., Gilbert, J.M., Stehle, T., Lencer, W., Benjamin, T.L., and Rapoport, T.A. (2003). Gangliosides are receptors for murine polyoma virus and SV40. EMBO J. *22*, 4346–4355.

29. Strayer, D., Branco, F., Zern, M.A., Yam, P., Calarota, S.A., Nichols, C.N., Zaia, J.A., Rossi, J., Li, H., Parashar, B., et al. (2002). Durability of transgene expression and vector integration: recombinant SV40-derived gene therapy vectors. Mol. Ther. *6*, 227–237.

30. Strayer, D.S. (1996). SV40 as an effective gene transfer vector in vivo. J. Biol. Chem. *271*, 24741–24746.

31. Liu, B., Daviau, J., Nichols, C.N., and Strayer, D.S. (2005). In vivo gene transfer into rat bone marrow progenitor cells using rSV40 viral vectors. Blood *106*, 2655–2662.

32. Strayer, D.S., Mitchell, C., Maier, D.A., and Nichols, C.N. (2010). Production of SV40-derived vectors. Cold Spring Harb. Protoc. *2010*, pdb.prot5436.

33. Gething, M.-J., and Sambrook, J. (1981). Cell-surface expression of influenza haemagglutinin from a cloned DNA copy of the RNA gene. Nature *293*, 620–625.

34. Asano, M., Iwakura, Y., and Kawade, Y. (1985). SV40 vector with early gene replacement efficient in transducing exogenous DNA into mammalian cells. Nucleic Acids Res. *13*, 8573–8586.

35. Zern, M.A., Ozaki, I., Duan, L., Pomerantz, R., Liu, S.L., and Strayer, D.S. (1999). A novel SV40-based vector successfully transduces and expresses an alpha 1-antitrypsin ribozyme in a human hepatoma-derived cell line. Gene Ther. *6*, 114–120.

36. Toscano, M.G., van der Velden, J., van der Werf, S., Odijk, M., Roque, A., Camacho-Garcia, R.J., Herrera-Gomez, I.G., Mancini, I., and de Haan, P. (2017). Generation of a Vero-based packaging cell line to produce SV40 gene delivery vectors for use in clinical gene therapy studies. Mol. Ther. Methods Clin. Dev. *6*, 124–134.

37. Kimchi-Sarfaty, C., Ben-Nun-Shaul, O., Rund, D., Oppenheim, A., and Gottesman, M.M. (2002). In vitro-packaged SV40 pseudovirions as highly efficient vectors for gene transfer. Hum. Gene Ther. *13*, 299–310.

38. Sauter, B.V., Parashar, B., Chowdhury, N.R., Kadakol, A., Ilan, Y., Singh, H., Milano, J., Strayer, D.S., and Chowdhury, J.R. (2000). A replication-deficient rSV40 mediates liver-directed gene transfer and a long-term amelioration of jaundice in gunn rats. Gastroenterology *119*, 1348–1357.

39. Louboutin, J.P., Marusich, E., Fisher-Perkins, J., Dufour, J.P., Bunnell, B.A., and Strayer, D.S. (2011). Gene transfer to the rhesus monkey brain using SV40-derived vectors is durable and safe. Gene Ther. *18*, 682–691.

40. Mueller, C., Strayer, M.S., Sirninger, J., Braag, S., Branco, F., Louboutin, J.-P., Flotte, T.R., and Strayer, D.S. (2010). In vitro and in vivo functional characterization of gutless recombinant SV40-derived CFTR vectors. Gene Ther. *17*, 227–237.

41. Sobrevals, L., Rodriguez, C., Romero-Trevejo, J.L., Gondi, G., Monreal, I., Pañeda, A., Juanarena, N., Arcelus, S., Razquin, N., Guembe, L., et al. (2010). Insulin-like growth factor I gene transfer to cirrhotic liver induces fibrolysis and reduces fibrogenesis leading to cirrhosis reversion in rats. Hepatology *51*, 912–921.

42. Maucksch, C., Connor, B., and Rudolph, C. (2013). Plasmid DNA concatemers: influence of plasmid structure on transfection efficiency. In Minicircle and Miniplasmid DNA Vectors: The Future of Nonviral and Viral Gene Transfer, M. Schleef, ed. (Wiley-VCH Verlag), pp. 59–69.

43. Vera, M., Prieto, J., Strayer, D.S., and Fortes, P. (2004). Factors influencing the production of recombinant SV40 vectors. Mol. Ther. *10*, 780–791.

44. Strayer, D.S., Lamothe, M., Wei, D., Milano, J., and Kondo, R. (2001). Generation of recombinant SV40 vectors for gene transfer. Methods Mol. Biol. *165*, 103–117.

45. Hastie, A.R., and Pruitt, S.C. (2007). Yeast two-hybrid interaction partner screening through in vivo Cre-mediated Binary Interaction Tag generation. Nucleic Acids Res. *35*, e141.

46. Zheng, T., Zhu, Z., Wang, Z., Homer, R.J., Ma, B., Riese, R.J., Jr., Chapman, H.A., Jr., Shapiro, S.D., and Elias, J.A. (2000). Inducible targeting of IL-13 to the adult lung causes matrix metalloproteinase- and cathepsin-dependent emphysema. J. Clin. Invest. *106*, 1081–1093.

47. Zhu, Z., Zheng, T., Lee, C.G., Homer, R.J., and Elias, J.A. (2002). Tetracycline-controlled transcriptional regulation systems: advances and application in transgenic animal modeling. *Semin Cell Dev Biol*,, *Volume 13* (Elsevier), pp. 121–128.

48. Zhu, Z., Ma, B., Homer, R.J., Zheng, T., and Elias, J.A. (2001). Use of the tetracycline-controlled transcriptional silencer (tTS) to eliminate transgene leak in inducible overexpression transgenic mice. J. Biol. Chem. *276*, 25222–25229.

49. Arad, U., Ben-Nun-Shaul, O., El-Latif, M.A., Nissim, O., and Oppenheim, A. (2002). A new packaging cell line for SV40 vectors that eliminates the generation of T-antigen-positive, replication-competent recombinants. Virology *304*, 155–159.

50. Strayer, D.S. (2000). Effective gene transfer using viral vectors based on SV40. In Gene Targeting Protocols. Methods in Molecular Biology, E.B. Kmiec, ed. (Humana Press), pp. 61–74.

51. Gluzman, Y. (1981). SV40-transformed simian cells support the replication of early SV40 mutants. Cell *23*, 175–182.

52. Meerbrey, K.L., Hu, G., Kessler, J.D., Roarty, K., Li, M.Z., Fang, J.E., Herschkowitz, J.I., Burrows, A.E., Ciccia, A., Sun, T., et al. (2011). The pINDUCER lentiviral toolkit for inducible RNA interference in vitro and in vivo. Proc. Natl. Acad. Sci. USA *108*, 3665–3670.

53. Seppen, J., Rijnberg, M., Cooreman, M.P., and Oude Elferink, R.P. (2002). Lentiviral vectors for efficient transduction of isolated primary quiescent hepatocytes. J. Hepatol. *36*, 459–465.

54. Peters, W.H., Allebes, W.A., Jansen, P.L., Poels, L.G., and Capel, P.J. (1987). Characterization and tissue specificity of a monoclonal antibody against human uridine 5'-diphosphate-glucuronosyltransferase. Gastroenterology *93*, 162–169.

OMTM, Volume *9*

Supplemental Information

Cre Recombinase Mediates the Removal of Bacterial Backbone to Efficiently Generate rSV40

Xiaoxia Shi, Matthew Ryan Ykema, Jaco Hazenoot, Lysbeth ten Bloemendaal, Irene Mancini, Machteld Odijk, Peter de Haan, and Piter J. Bosma

Figure S1

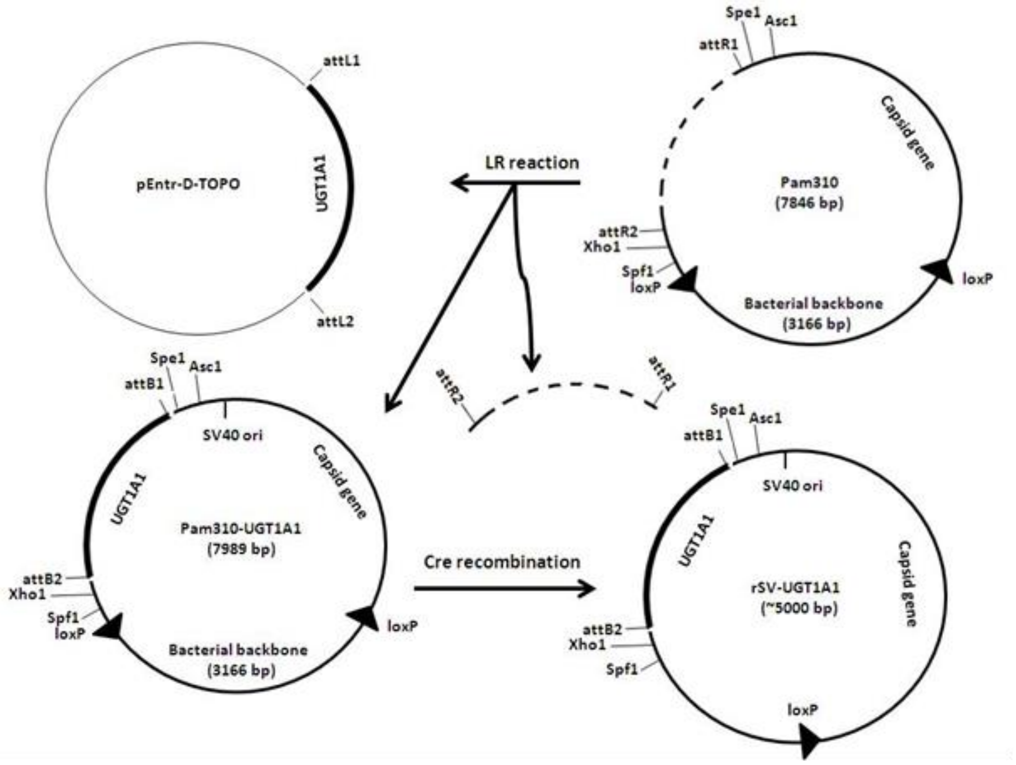

Figure S1. Construction of rSV-*hUGT1A1* vector. The human hUGT1A1 coding region was amplified, cloned to pEntr-d-TOPO vector and sequenced to exclude mutations. The resulting plasmid was used as the donor vector to insert the hUGT1A1 gene in the pAM310 destination vector by LR reaction[1].

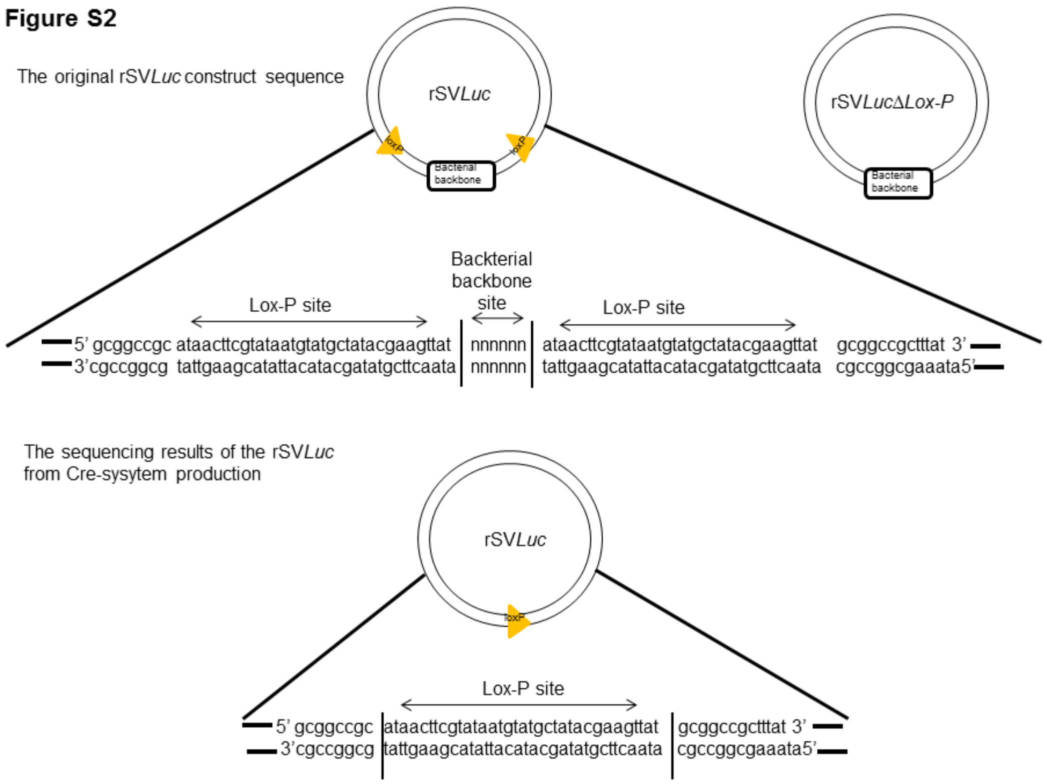

Figure S2. PCR amplification to confirm Cre mediated removal of the bacterial backbone from rSV*Luc*. For rSV-*hUGT1A1* and rSV*GFP* the same approach was used.

Figure S3

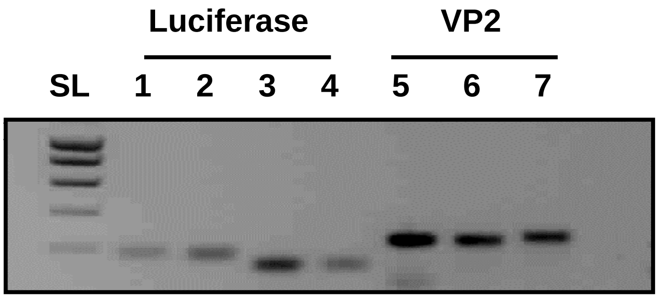

Figure S3. DIG-labeled PCR products. *Lane 1*, luciferase_1; *Lane 2*, luciferase_2; *Lane 3*, luciferase_3; *Lane 4*, luciferase_4; *Lane 5*, VP2_1; *Lane 6*, VP2_2; *Lane 7*, VP2_3.

Table S1

**Ampicillin standard curve**		**Virus**	
**log number**	**Ct value**	**batches**	**Ct value**
**0**	**32,018**	**rSV*Luc1***	**32,23839**
**1**	**29,307**	**rSV*Luc2***	**34,20386**
**2**	**26,289**	**rSV*Luc3***	**32,77787**
**3**	**23,722**	**rSV*Luc4***	**32,61454**
**4**	**20,092**		
**5**	**17,527**		
**6**	**15,348**		

Supplemental reference

1. Toscano, MG, van der Velden, J, van der Werf, S, Odijk, M, Roque, A, Camacho-Garcia, RJ, *et al.* (2017). Generation of a Vero-based packaging cell line for the production of SV40 gene delivery vector particles for use in clinical gene therapy studies. *Molecular Therapy-Methods & Clinical Development*.
